# CAV-2-Mediated GFP and LRRK2^G2019S^ Expression in the *Macaca fascicularis* Brain

**DOI:** 10.3389/fnmol.2020.00049

**Published:** 2020-03-25

**Authors:** Carla di Caudo, Ivan Martínez-Valbuena, Iñaki-Carril Mundiñano, Aurelie Gennetier, Maria Hernandez, Mar Carmona-Abellan, Irene Marcilla Garcia, Eric J. Kremer, Rosario Luquin

**Affiliations:** ^1^Division of Neuroscience, Center for Applied Medical Research (CIMA), Universidad de Navarra, Pamplona, Spain; ^2^Department of Neurology, Clinica Universidad de Navarra, Pamplona, Spain; ^3^Instituto de Investigación Sanitaria de Navarra (IDISNA), Pamplona, Spain; ^4^Institut de Génétique Moléculaire de Montpellier, Université de Montpellier, CNRS, Montpellier, France

**Keywords:** *M. fascicularis*, CAV-2, viral vectors, GFP, LRRK2, Parkinson’s disease, nonhuman primate, CNS

## Abstract

Parkinson’s disease is characterized by motor and nonmotor symptoms that gradually appear as a consequence of the selective loss of dopaminergic neurons in the *substantia nigra pars compacta*. Currently, no treatment can slow Parkinson’s disease progression. Inasmuch, there is a need to develop animal models that can be used to understand the pathophysiological mechanisms underlying dopaminergic neuron death. The initial goal of this study was to determine if canine adenovirus type 2 (CAV-2) vectors are effective gene transfer tools in the monkey brain. A second objective was to explore the possibility of developing a large nonhuman primate that expresses one of the most common genetic mutations causing Parkinson’s disease. Our studies demonstrate the neuronal tropism, retrograde transport, biodistribution, and efficacy of CAV-2 vectors expressing GFP and leucine-rich repeat kinase 2 (LRRK2^G2019S^) in the *Macaca fascicularis* brain. Our data also suggest that following optimization CAV-2-mediated LRRK2^G2019S^ expression could help us model the neurodegenerative processes of this genetic subtype of Parkinson’s disease in monkeys.

## Introduction

Parkinson’s disease is a complex neurodegenerative disorder clinically characterized by a triad of motor symptoms, including tremor, rigidity, and bradykinesia (Poewe et al., 2017). Approximately 9 million individuals are directly affected by Parkinson’s disease, making it the second most prevalent neurodegenerative disease among the elderly. The etiology and pathogenic mechanisms of Parkinson’s disease remain poorly understood with only a small proportion having an identifiable monogenic cause (with an overall prevalence lower than 5%; Poewe et al., 2017). Among the genetic causes, autosomal dominant mutations in PARK8, the gene coding for leucine-rich repeat kinase 2 (LRRK2), are the most common cause of familial Parkinson’s disease (Paisán-Ruíz et al., 2004; Zimprich et al., 2004). LRRK2 is a 285 kDa multifunctional protein that contains several predicted domains including a serine/threonine kinase domain, a GTPase domain and putative protein-protein interaction domains (Gloeckner et al., 2006; Smith et al., 2006; Guo et al., 2007; Ito et al., 2007; Li et al., 2007). LRRK2 is expressed in brain areas receiving dopaminergic innervation, such as the striatum, hippocampus, cortex, and cerebellum, while lower levels have been reported in neurons of the *substantia nigra* (SN) and ventral tegmental area (Taymans and Baekelandt, 2014). In neurons, LRRK2 plays a role in neurogenesis, axonal outgrowth, mitochondrial function, autophagy, and synaptic function (Shin et al., 2008; Matta et al., 2012; Wang et al., 2012; MacLeod et al., 2013; Sepulveda et al., 2013; Godena et al., 2014; Law et al., 2014; Sweet et al., 2015). At least six mutations in LRRK2 are pathogenic. A glycine to serine change at amino acid 2019 (LRRK2^G2019S^) accounts for ~7% of familial cases and ~2% of sporadic, late-onset, cases (Gasser et al., 2011). LRRK2^G2019S^ is thought to have modestly increased kinase activity, which, by an unknown mechanism, induces neuronal and non-neuronal cell loss (Mortiboys et al., 2015).

Several LRRK2^G2019S^ rodent models have been generated with the goal of better understanding the molecular pathogenesis of Parkinson’s disease. These models include knock-out (KO; Andres-Mateos et al., 2009; Lin et al., 2009; Tong et al., 2010, 2012; Hinkle et al., 2012; Ness et al., 2013) or knock-in (KI; Tong et al., 2009; Yue et al., 2015), bacterial artificial chromosome (BAC)-mediated transgenesis (Li et al., 2009, 2010; Melrose et al., 2010; Winner et al., 2011; Bichler et al., 2013; Lee et al., 2015; Sweet et al., 2015), non-BAC transgenics (Chen et al., 2012; Chou et al., 2014; Walker et al., 2014), and those with temporally-controlled or inducible LRRK2 expression (Lin et al., 2009; Lee et al., 2010; Dusonchet et al., 2011; Zhou et al., 2011; Walker et al., 2014; Tsika et al., 2015). The data from LRRK2 KO mice suggest that LRRK2 plays little, if any, role in the development or maintenance of murine dopaminergic neurons (Li et al., 2010; Melrose et al., 2010). In addition, KI LRRK2^G2019S^ mice have minimal reduction in dopamine (DA) release, axonal pathology, and/or evidence of neurodegeneration (Li et al., 2010; Melrose et al., 2010). The reasons why rodents do not exhibit substantial pathology are still uncertain, but not unusual—many rodent models poorly recapitulate human neurodegenerative diseases. In the case of BAC and KI mice, LRRK2 is expressed during development and therefore compensatory mechanisms may prevent the loss of DA neurons (Dawson et al., 2010) and the appearance of motor symptoms. Thus, alternative models are needed to reproduce the progressive degeneration of nigral neurons associated with the LRRK2^G2019S^.

A handful of studies using vector-mediated delivery of LRRK2^G2019S^ to the rodent brain reported a modest loss of dopaminergic cells without changes in motor performance (Lee et al., 2010; Dusonchet et al., 2011). These studies used herpes simplex virus (HSV) or human adenovirus 5 (HAdV-C5) vectors, but did not target SN neurons and/or allow long-term transgene expression (Dai et al., 1995; Silva et al., 2010; Lentz et al., 2012). For gene transfer to neurons in the CNS, canine adenovirus type 2 (CAV-2) vectors are particularly interesting: in rodents, dogs, and prosimians, the vectors preferentially transduce neurons and are efficiently transported from the injection site to efferent regions (Soudais et al., 2001; Cubizolle et al., 2013; Junyent and Kremer, 2015; Mestre-Francés et al., 2018). Helper-dependent (HD) CAV-2 vectors, which are devoid of all viral coding sequences, also lead to long-term expression in the CNS (del Rio et al., 2019). In the case of LRRK2 expression, the ~8 kb cDNA is challenging to express from some viral vectors. Of note though, HD CAV-2 vectors are an ideal option because they can harbor an expression cassette as large as 30 kb (Soudais et al., 2004). We previously showed that injections of HD-LRRK2^G2019S^, an HD CAV-2 vector containing a leucine-rich repeat kinase 2^G2019S^ expression cassette, in the striatum of the gray mouse lemur (*Microcebus murinus*) induces Parkinson’s disease-like histological lesions and motor symptoms (Mestre-Francés et al., 2018; Lasbleiz et al., 2019). As a prelude to the development of a monkey with Parkinson’s disease, CAV-2 vector efficacy studies in large brains need to be documented. Here, our study using CAV-2 vectors expressing GFP and LRRK2^G2019S^ demonstrate the neuronal tropism, retrograde transport, efficient biodistribution, and efficacy following injections in monkey striatum and the SN.

## Materials and Methods

### Animals

Experimental protocols were carried out under a project license according to the European Communities Council Directive of 24/11/1986 (86/609/EEC) regarding the care and use of animals for experimental procedures and under the guidance of the Ethics Committee for Animal Experimentation of the University of Navarra. Twenty, 4–5-year-old, male, 3–5 kg, *M. fascicularis* were included in the study. Animals were housed in a facility under standard conditions of air exchange (16 l/min), humidity (50%), and light/night cycles, and were fed fresh fruit and commercial pellets, with free access to water.

### CAV-2 Vectors

CAV-GFP (Kremer et al., 2000), HD-GFP (Soudais et al., 2004), and HD-LRRK2^G2019S^ (Mestre-Francés et al., 2018) have been previously described. CAV-GFP is a replication-defective E1-deleted vector harboring a cytomegalovirus early promoter (CMV), GFP, SV40 polyA cassette. HD-GFP is a HD CAV-2 vector expressing GFP. HD-LRRK2^G2019S^ contains a Rous sarcoma virus (RSV) promoter, a codon-optimized LRRK2^G2019S^ cDNA, followed by an internal ribosomal entry site (IRES), GFP, and an SV40 polyA. This cassette was initially cloned into pGut3 and then inserted into a pEJK25 *via* homologous recombination to create pHD-LRRK2^G2019S^. HD-LRRK2^G2019S^ was amplified and purified similar to that used for HD-GFP with minor modifications (Cubizolle et al., 2013). The HD-GFP stock used during this study was 1.3 × 10^12^ pp/ml with an infectious particle to a physical particle ratio of 1:10. The HD-LRRK2^G2019S^ stocks used during this study were 3–7 × 10^11^ pp/ml with an infectious particle to a physical particle ratio of ~1:25.

### Injections

Four monkeys received CAV-GFP injections and were killed 1 month postinjection to assess safety and biodistribution of the CAV-2 in the brain: two of these four monkeys received an injection in the left putamen, and the other two monkeys received bilateral injections in the SN. In one of the latter monkeys, the injections missed the target and therefore was not included in the analyses. Eight monkeys received injections of HD-LRRK2^G2019S^: four of the eight monkeys received an injection into the left putamen and were killed as planned 6 months postinjection; and the remaining four monkeys received injections in the SN. One monkey in the latter cohort died due to intracranial hemorrhage at 15 days postinjection. Also, four monkeys received injections of HD-GFP into the left SN. One monkey died due to heart failure. The six remaining monkeys were killed 12 months postinjection. As the neuropathological changes observed 6 months postinjection in the monkeys injected in the putamen with the HD-LRRK2^G2019S^ were sparse, a different timepoint of 12 months postinjection was used for those monkeys that received a nigral injection, to try to induce more robust neuropathological changes. A cohort of four non-injected (intact) animals were used as a control for histological analyses.

### Surgery

Stereotaxic surgery was performed according to the coordinates for stereotaxic injections based upon MRI guidance and ventriculography. Before surgery, a brain MRI was performed in each animal under light sedation with an intramuscular injection of ketamine (10 mg/kg) and midazolam (0.5 mg/kg) and. Initial coordinates for injection sites were ascertained using the Osirix Medical Image Software (version 3.9.1). On the day of surgery, the monkeys were anesthetized by intramuscular injections of ketamine and midazolam at the same doses above mentioned. Supplementary doses were given during surgery if necessary. The animals were placed in the stereotaxic frame and the vector delivery was performed following the convection-enhanced delivery (CED) method using an infusion pump (KDS200, LabNet Biotecnica, Madrid, Spain) at a rate of 1 μl/min the first 10 μl, 1.5 μl/min until 20 μl and 2 μl/min until 30 μl. CAV-GFP and HD-LRRK2^G201S^ were injected in 60 μl [1 × 10^10^ physical particles (pp)] of the corresponding vector divided in two 30 μl injections in left putamen (target 1: *X* = 10, *Y* = +1, *Z* = +1 and target 2: *X* = 12, *Y* = 4, *Z* = +3). A volume of 10 μl with 1 × 10^10^ pp of the corresponding vector was injected in each SN in CAV-GFP and left SN in HD-LRRK2^G2019S^ and HD-GFP groups (target: *X* = 4, *Y* = −8, *Z* = −4). These volumes were used based on the relative size of the structure being targeted and the advice and collective results from the Bankiewicz lab (UCSF). Coordinates were derived from Martin and Bowden (1996).

### PET Scans With ^11^C- DTBZ and ^18^F-FDG Ligands

Positron emission tomography (PET) with ^11^C-(+)-α-dihydro-tetrabenazine (DTBZ; used to quantify the nigrostriatal terminal density) and with ^18^F-deoxyglucose (FDG; to evaluate the glucose metabolism) were performed as previously described (Blesa et al., 2010).

### Transcardiac Perfusion and Tissue Preparation

After an overdose of a mixture of ketamine and midazolam, animals were transcardially perfused with 0.01 M PBS/4% paraformaldehyde (PFA, Sigma–Aldrich, St. Louis, MO, USA). Brains were immediately removed, blocked using a monkey brain matrix (ASI Instruments, Warren, MI, USA) and post-fixed for 2 days in 4% PFA/PBS. The brains were then cryoprotected in a 30% sucrose (Sigma–Aldrich, St. Louis, MO, USA) solution in 0.01 M PBS until processing. Brains were sliced into 40-μm-thick coronal sections along the rostral axis with a freezing microtome (SM 2000R, Leica, Germany) and collected in 0.125 M PBS containing 2% dimethylsulphoxide (Sigma–Aldrich, St. Louis, MO, USA), 20% glycerine (Panreac, Barcelona, Spain) and 0.05% sodium azide (Sigma–Aldrich, St. Louis, MO, USA) and were stored at −20°C until ulterior analysis.

### DNA Extraction and qPCR

Total DNA was extracted from fifteen 40-μm-thick PFA-fixed sections from the putamen, motor cortex and the SN of each animal with the QIAamp DNA FFPE Kit (Qiagen, Gaithersburg, MD, USA) and according to manufacturer’s protocol (without the deparaffinization step). Right and left hemispheres were analyzed separately. DNA integrity was confirmed by electrophoresis, and its concentration and purity assessed spectrophotometrically. CAV-2 vector genomes were then quantified by PCR (qPCR) using the inverted terminal repeats (ITR) sequences. To detect the amplification products, qPCR was performed on these DNAs with Power SYBR^®^ Green (Applied Biosystems, Foster City, CA, USA) and specific primers using an ABI Prism 7300 sequence detector (Applied Biosystems, Foster City, CA, USA). The primer sequences used for qPCR were forward: AGGACAAAGAGGTGTGGCTTA; reverse: GAACTCGCCCTGTCGTAAAA. The samples were run in triplicate. Data were normalized against the GAPDH sequence amplified using the primers GAPDH forward: CCACCCAGAAGACTGTGGAT; GADPH reverse: TTCAGCTCAGGGATGACCTT. Reference curves were established by determining cycle threshold (Ct) values for the amplification of serial dilutions of the CAV-2 genome.

### Histology

Immunohistochemistry (IHC) and immunofluorescence (IF) staining were performed on free-floating sections (see Table 1 for primary antibodies and Table 2 for secondary antibodies). Tissue sections were washed in bi-distilled water and 0.01 M PBS to remove the cryoprotectant solution and incubated in PBS with 0.02% hydrogen peroxide (H_2_O_2_; Merck Millipore, Darmstadt, Germany) for endogenous peroxidase inhibition. After that, they were blocked in 5% normal serum (goat or donkey in the function of the secondary antibody used) with 0.2% Triton X-100 (Sigma–Aldrich, St. Louis, MO, USA) for 60 min and then incubated overnight in the same solution containing a primary antibody. After being rinsed in 0.01 M PBS, tissue sections were incubated in 0.01 M PBS with 5% normal serum and containing the corresponding secondary antibody. The type of antibody, the time of incubation and subsequent steps were dependent on IHC or IF technique performed. In the IHC, the sections were incubated at room temperature with the corresponding biotin secondary antibody for 30 min and after, the sections were rinsed with the vector avidin-biotin complex (1:200 Vectastain ABC kit, Vector Laboratories, Burlingame, CA, USA) for 30 min. Staining for peroxidase was performed with the DAB substrate kit (Vector Laboratories, Burlingame, CA, USA). Finally, the sections were rinsed in double-distilled water and 0.01 M PBS, mounted on gelatin-coated slides and air-dried. The next day, almost all the sections were Nissl counterstained and coverslipped using DPX (VWR, Radnor, PE, USA). In the IF, the corresponding secondary antibody was incubated for 2 h in 0.01 M PBS containing normal donkey/goat serum (1:20). Finally, some sections were counterstained with a nucleic acid stain (TO-PRO-3 iodide, Molecular Probes, Waltham, MA, USA) and coverslipped with mounting medium (Immu-mount, Thermo-Shandon).

**Table 1:** Primary antibodies used in the study.

Antigen	Host	Source, catalog	Working concentration/Incubation period (h)	Normal localization
GFP	Rabbit polyclonal	Molecular Probes, A11122 RRID:AB_221569	1:1,000/16	Recognizes the CAV-GFP/ HD-GFP transduced areas
MTCO2	Mouse monoclonal	Abcam, AB3298 RRID:AB_303683	1:500/24	Recognizes mitochondria
NeuN	Mouse monoclonal	Millipore, MAB377 RRID:AB_2298772	1:1,000/24	Neurons
PHF-1	Rabbit polyclonal	Calbiochem, 577815	1:500/24	Recognizes the phospho-Tau epitope (^Ser396/Ser404^)
Tyrosine Hydroxylase (TH)	Mouse monoclonal	Millipore, MAB5280 RRID:AB_2201526	1:1,000/16	Dopaminergic neurons
TH	Rabbit polyclonal	Millipore, AB152 RRID:AB_390204	1:1,000/16	Dopaminergic neurons
VMAT2	Rabbit polyclonal	Novus Biologicals, NBP1-69750 RRID:AB_11035444	1:500/16	Monoaminergic neurons

**Table 2:** Secondary antibodies used in the study.

Antigen	Host	Source, catalog	Working concentration/Incubation period (h)
Anti-mouse Alexa 546	Goat	Molecular Probes, A11003 RRID:AB_141370	1:500/2
Anti-mouse Alexa 568	Donkey	Molecular Probes, A10037 RRID:AB_2534013	1:500/2
Anti-mouse Alexa 488	Goat	Molecular Probes, A11029 RRID:AB_138404	1:500/2
Anti-mouse Alexa 488	Donkey	Molecular Probes, A21202 RRID:AB_141607	1:500/2
Anti-rabbit Alexa 555	Donkey	Molecular Probes, A31572 RRID:AB_162543	1:500/2
Anti-rabbit Alexa 568	Goat	Molecular Probes, A11011 RRID:AB_143157	1:500/2
Anti-rabbit Alexa 488	Goat	Molecular Probes, A11034 RRID:AB_2576217	1:500/2
Biotin anti-mouse	Goat	Dako, E0433012 RRID:AB_2687905	1:200/0.5
Biotin anti-rabbit	Goat	Dako, E0432 RRID:AB_2313609	1:200/0.5

### Cell Counting and Volume Measurement

The total number of TH^+^ and VMAT2^+^ neurons in the SN were quantified according to the optical fractionator principle (Olympus CAST system, Denmark). Cells in every 14th section were quantified. To determine the area transduced following CAV-GFP injection in the putamen, 40-μm-thick coronal sections were generated and anti-GFP IHC and Nissl counterstaining was performed. Every 12th section, from +7 to −8 (according to the Martin and Bowden atlas) was used. The striatal volume (caudate + putamen) and the volume of GFP-immunoreactivity were quantified according to the Cavalieri principle using CAST software. The transduced area in SN in the animals of the CAV-GFP group was determined using stereology in consecutive serial coronal sections of SN stained using anti-TH and anti-GFP IHC.

### Densitometry of pTau Immunoreactivity

Samples were viewed and digitalized with an Olympus BX-51 microscope equipped with an Olympus DP-70 camera using the CAST grid software package (Olympus, Denmark). The images were analyzed using ImageJ software, converted to an 8-bit (binary) format and the background (20 pixels) subtracted. Consequently, the perimeter of each layer was outlined manually for each image excluding any unwanted immune stained structures (i.e., capillary). Then, the same threshold limits were defined for each image and the percentage of immune-reactive area was determined. The images were obtained from different brain regions and different magnification according to the protein in the study. IHC was performed using the same incubation times. The same investigator performed all quantifications with masked sections.

### Statistical Analyses

The histological findings (volume, stereology, and densitometry) were analyzed by parametric tests by comparing intact and animals injected with HD-LRRK2^G2019S^ in the putamen, whereas the comparisons between SN-injected HD-GFP/LRRK2^G2019S^ animals were performed with non-parametric analysis. Finally, comparisons between different groups were performed using the Kruskal–Wallis test followed by the Mann–Whitney test (2–2). The results are expressed as a mean ± standard error of the mean (SEM).

## Results

### CAV-2 Preferentially Infects Neurons and Is Transported to Efferent Regions

**Figure 1:**
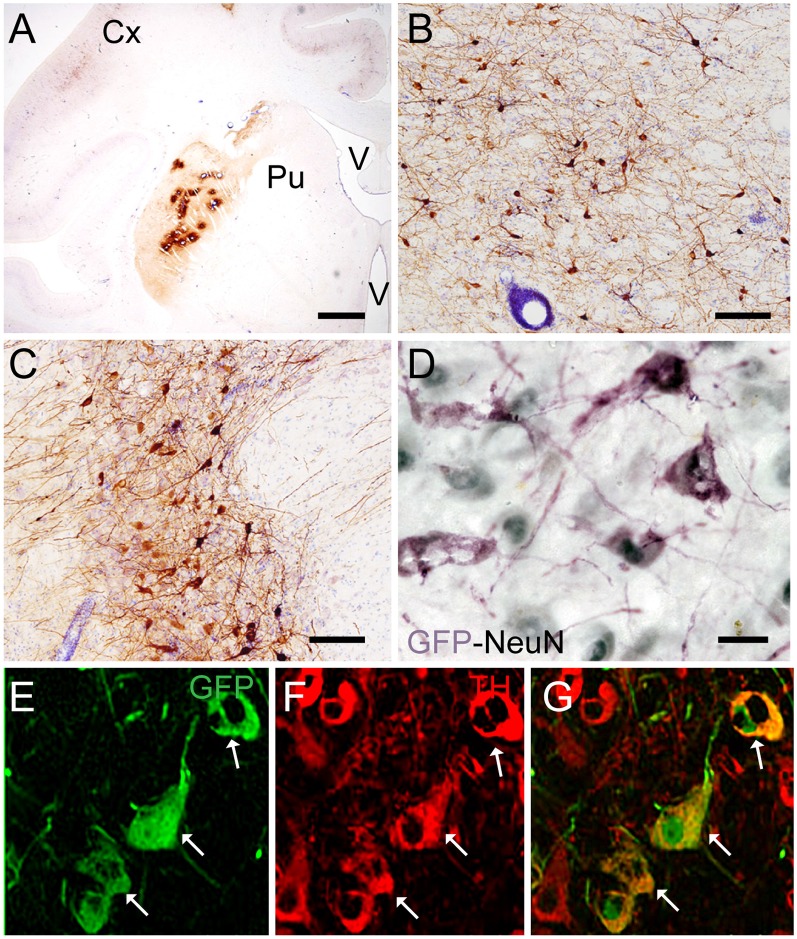
Coronal sections of animals injected with canine adenovirus type (CAV)-GFP in the left putamen. **(A)** Low magnification of immunohistochemistry (IHC) against GFP and Nissl counterstained of the injected putamen; **(B)** higher magnification of IHC against GFP and Nissl counterstained of the injected thalamus; **(C)** IHC against GFP and Nissl counterstained of the substantia nigra (SN) of the injected hemisphere; **(D)** IHC against GFP (pink) and NeuN (black) in the SN of the injected hemisphere; and in the contralateral SN **(E)** immunofluorescence (IF) against GFP (green; **F**) IF against TH (red; **G**) merge of **(E,F)**. White arrows denoted TH^+^/GFP^+^ cells. Scale bars: **(A)** 1 mm; **(B,C)** 100 μm; **(D)** 10 μm; **(E)** 5 μm.

Targeting the putamen in the monkey brain is straightforward and allows one to rapidly determine the efficacy of vector transduction and retrograde transport, due to its multiple and well-characterized connections. We, therefore, injected CAV-GFP, a replication-defective CAV-2 vector harboring a GFP expression cassette, in the *M. fascicularis* left putamen. GFP was observed within cell bodies and projections with neuron-like morphology (Figures 1A–G). The GFP signal in the putamen and caudate (Figure 1A) corresponds to a volume of 540 mm^3^ (~47% of total striatum (1,140 mm^3^), with an approximative ratio between injected volume and transduced volume of 1:9. We also detected GFP expression in the soma of cells located in the injected and contralateral motor cortex, claustrum, parafascicularis, centromedian thalamus nuclei, and in the SN, where the majority were NeuN^+^ (Figure 1D). We also quantified the efficacy of retrograde transport from the putamen to the SN. In this pilot assay, ~5% of the ~1,20,000 TH^+^ cells neurons in the ipsilateral SN were GFP^+^. Of note, we also found a handful of GFP^+^/TH^+^ cells in the contralateral SN (Figures 1E–G). Together, these data demonstrate CAV-2 vector infection at the site of injection, the retrograde transport from the putamen to efferent regions, and that the monkey SN sends DA projection to the striatum in each hemisphere.

### Injections of HD-LRRK2G2019S in the Putamen

We then explored the injection of HD-LRRK2^G2019S^ (Mestre-Francés et al., 2018) in the *Macaca fascicularis* brain. Because dopaminergic neurons in the SN are lost in Parkinson’s disease patients, our null hypothesis was that one may need to target these neurons to induce disease-like features. Notably, striatal injection of CAV-2 vectors leads to a transduction efficacy of ~70% of the dopaminergic neurons in the SN of the gray mouse lemur (Mestre-Francés et al., 2018). While the injection of CAV-GFP in the monkey putamen suggested that technical improvements would be needed to reach this level, a non-cell autonomous effect of LRRK2^G2019S^ activity might impact the putamen and/or SN (di Domenico et al., 2019). We, therefore, injected four monkeys in the left putamen with HD-LRRK2^G2019S^. Of note, in the gray mouse lemur, we found no adverse physiological, histological, or biological effects from a control vector (HD-GFP) injections. Nonetheless, because a cohort of monkeys injected with HD-GFP was not performed at this stage, the below results are only suggestive.

**Figure 2:**
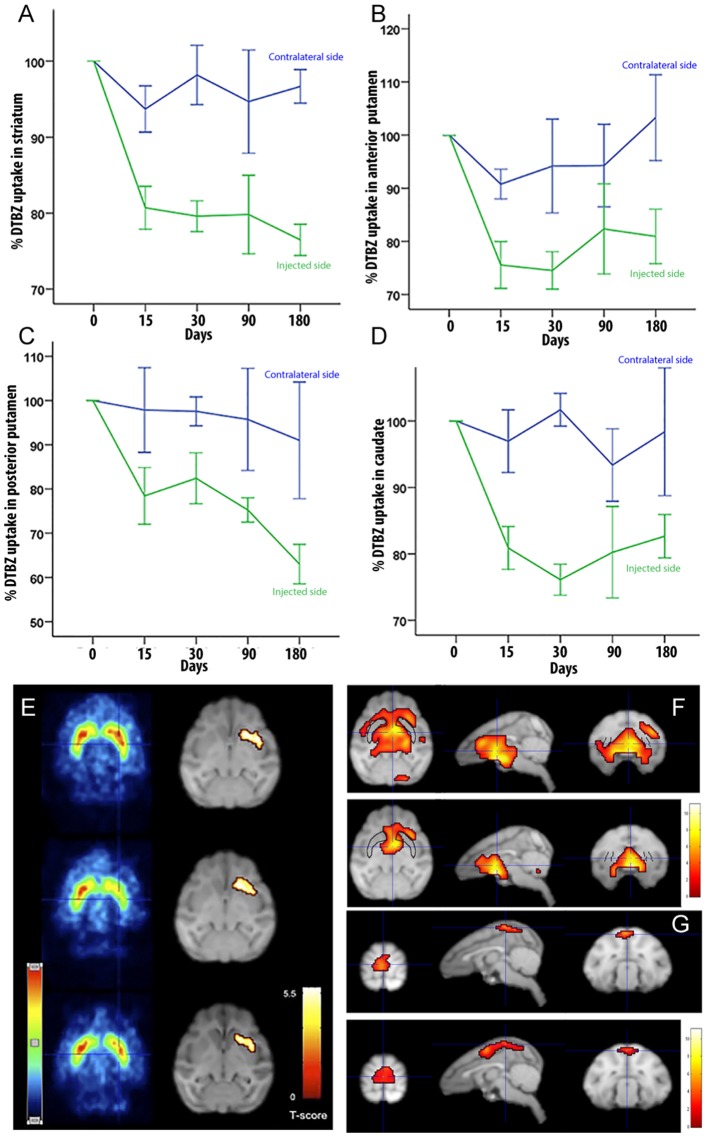
Quantification of DTBZ and FDG uptake *via* PET scan following putamen injections of helper-dependent (HD)-LRRK2^G2019S^. **(A)** Representative image of DTBZ-PET scan in the striatum; **(B)** anterior putamen; **(C)** posterior putamen; and **(D)** caudate. SPM analysis of the FDG PET scan showing **(E)** reduction in the hypermetabolic areas. SPM analyses suggested a change in dopaminergic uptake (*p* ≤ 0.001) in the left striatum that remained stable at 0.5, 3, and 6 months after surgery right column; and **(F–G)** changes in the hypometabolic areas.

To determine if LRRK2^G2019S^ activity could influence nigrostriatal termini density or DA uptake in the striatum, we measured ^11^C-DTBZ and ^18^F-FDG levels by PET. Compared to pre-injection levels, we found a decrease in DTBZ uptake throughout the injected striatum at 15 days postinjection, which remained stable during the 6-months follow-up (Figure 2A). When the anterior and posterior striatum were analyzed separately, we found that DTBZ uptake was reduced during the first month in the anterior putamen (Figure 2B), and a progressive reduction in the posterior putamen (Figure 2C). DTBZ uptake in the caudate was relatively stable (Figure 2D). FDG-PET scans at 0.5, 3, and 6 months postinjection showed bilateral hypermetabolism in the ventral striatum, thalamus and midbrain at 6 months (Figures 2E–G). Quantification suggested that the ipsilateral pre-frontal gyro, bilateral superior frontal gyro, and bilateral thalamus displayed hypermetabolism 3 months postinjection. We also noted a trend towards a mild, bilateral, parieto-occipital hypometabolism (Figure 2G).

**Figure 3:**
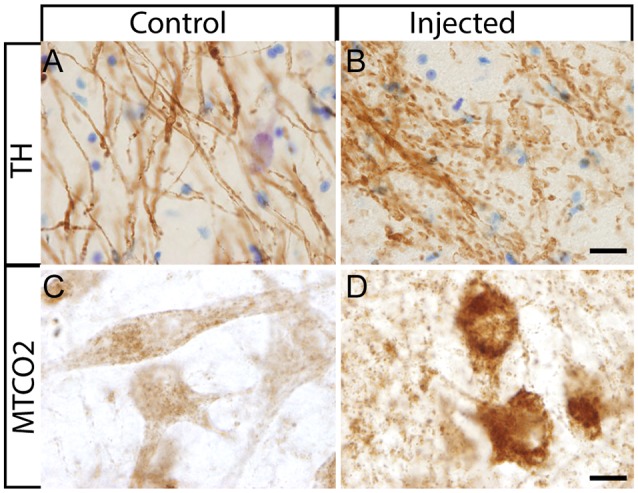
Histological aspect of nigral neurons after the injection of HD-LRRK2^G2019S^ in the putamen.** (A,B)** IHC against TH and Nissl counterstained in the SN from an intact monkey and in the SN from a monkey injected in the putamen. Dystrophic neurites (broken and swollen axons) in the injected hemisphere. **(C,D)** IHC against mitochondrially-encoded cytochrome C oxidase II (MTCO2) in nigral neurons of an intact monkey and a representative image from an HD-LRRK2G2019S injected monkey. Scale bars = 20 μm.

**Figure 4:**
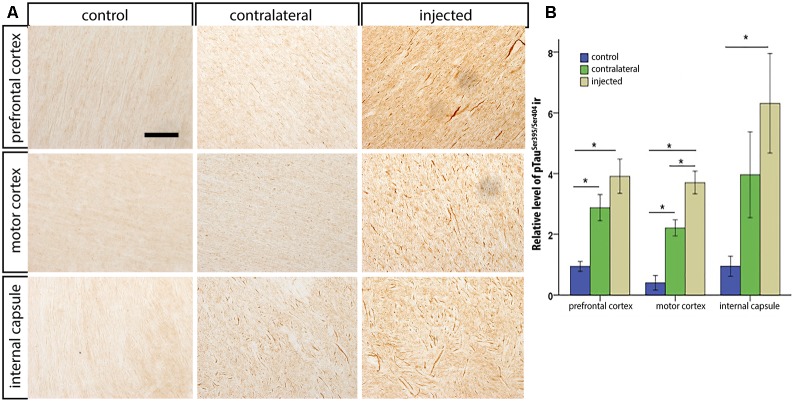
pTau^Ser395/Ser404^ immunoreactivity following HD-LRRK2^G2019S^ injections in the putamen: pTau^Ser395/Ser404^ immunoreactivity in the internal capsule in **(A)** an intact animal (control), contralateral hemisphere, and injected hemisphere. Scale bar, 100 μm. **(B)** Quantification of pTau^Ser395/Ser404^ immunoreactivity by optic densitometry. *p ≤ 0.05 (student’s *t*-test).

After the *in vivo* follow-up, the monkeys were killed, the brains fixed, and prepared for downstream assays. Initially, we found no difference in the number of TH^+^ cells between the injected vs. the contralateral hemisphere (1,04,000 ± 24,000 vs. 1,14,000 ± 22,000, respectively), or in the number of VMAT^+^ cells (1,14,000 ± 22,400 vs. 1,19,000 ± 14,000, respectively). In contrast to the contralateral hemisphere, some SN cells in the injected hemisphere presented with dystrophic neurites, and broken and swollen axons (Figures 3A,B). Also, the somas of some nigral neurons were fusiform and more mitochondrially-encoded cytochrome C oxidase II (MTCO2) immunoreactive (Figures 3C,D). The presence of pTau^Ser395/Ser404^ in cortex regions is a common feature in LRRK2 model systems (MacLeod et al., 2006; Li et al., 2009; Melrose et al., 2010) and 79% of LRRK2 mutation carriers have been reported to have tau pathology (Poulopoulos et al., 2012). We, therefore, assayed for pTau^Ser395/Ser404^ accumulation. We found increased IR in the white matter of the prefrontal, motor cortex and internal capsule of the injected vs. that from contralateral hemisphere and intact animals (Figures 4A,B). Finally, we used brain sections from the SN, putamen and motor cortex to isolate total DNA. Using qPCR targeting a conserved part of each vector sequence, we detected genomes in both hemispheres, with levels consistently higher in the injected hemisphere (Table 3).

**Table 3:** Vector genomes in the HD-LRRK2^G2019S^ putamen-injected cohort.

Left hemisphere (injected)			Right hemisphere		
SN	putamen	cortex	SN	putamen	cortex
7,070 (±2,320)	23,100 (± 8,960)	1,530 (± 270)	1,190 (± 390)	4,080 (± 325)	637 (± 90)

Together, these data suggest but do not unequivocally demonstrate, that expression of LRRK2^G2019S^ induces some of the histopathological hallmarks present in patients with genetic Parkinson’s disease. Although HD-CAV-2 vectors lead to long-term transgene expression *in vivo*, and that it is unlikely that capsid uptake still had an effect on neurons 6 months postinjection (Piersanti et al., 2015; Mestre-Francés et al., 2018; del Rio et al., 2019), further controls need to be performed to show that these phenotypes are not linked to the CAV-2 capsid.

### CAV-GFP, HD-GFP, and HD-LRRK2G2019S Injections Into the SN

**Figure 5:**
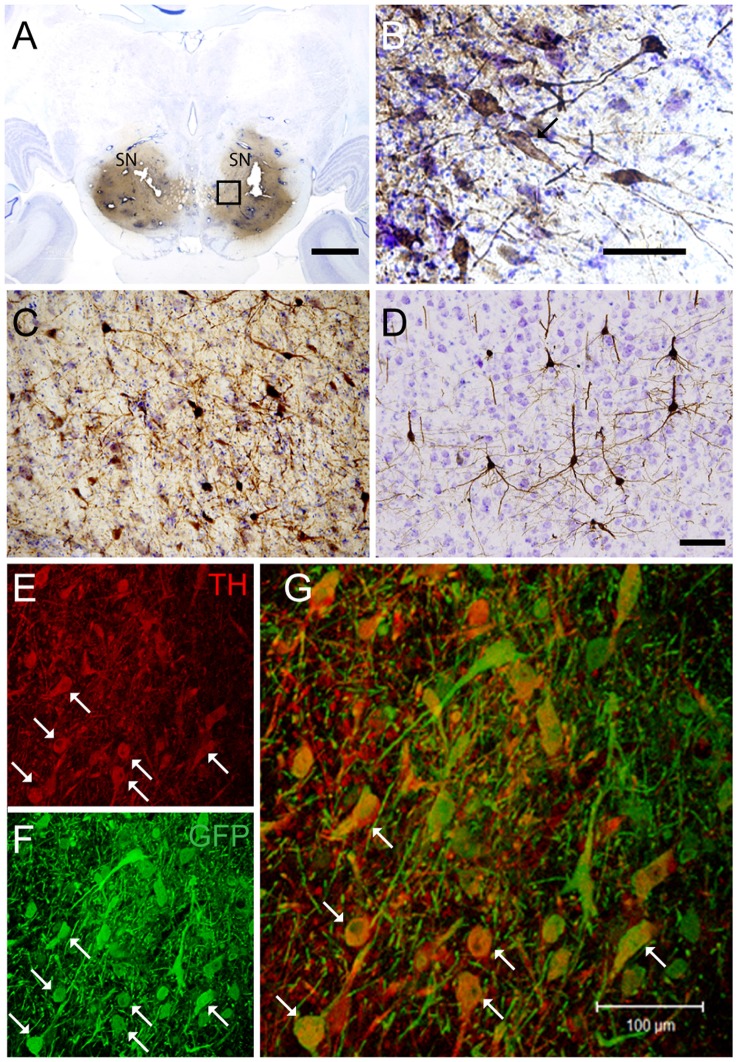
CAV-GFP injection in macaque SN. Coronal sections of animals injected bilaterally in the SN. **(A)** Low magnification of IHC against GFP and Nissl counterstained of an injected SNs; **(B)** higher magnification of GFP expression and Nissl counterstaining in the SN; and **(C)** GFP-IR in the pedunculopontine tegmental nucleus; **(D)** GFP-IR in the motor cortex; **(E,F)** IF against GFP (green) and TH (red) in an SN **(G)** merge of **(E,F)**. White arrows denoted TH^+^/GFP^+^ cells. Scale bars **(A)** 5 mm; **(B–D)** 100 μm; **(E)** 20 μm.

If a cell-autonomous effect of LRRK2^G2019S^ is responsible for the loss of SN neurons in Parkinson’s disease, then gene transfer needs to be more efficient in these cells. Therefore, in the second set of pilot assays, we bilaterally injected CAV-GFP in the SN to test vector efficacy. GFP^+^ neurons were detected in the SN, motor cortex, putamen, caudate, lateral hypothalamus, and the pedunculopontine nucleus bilaterally (Figures 5A–G). Of note, ~72% of the TH^+^ cells in SNs were GFP^+^.

**Figure 6:**
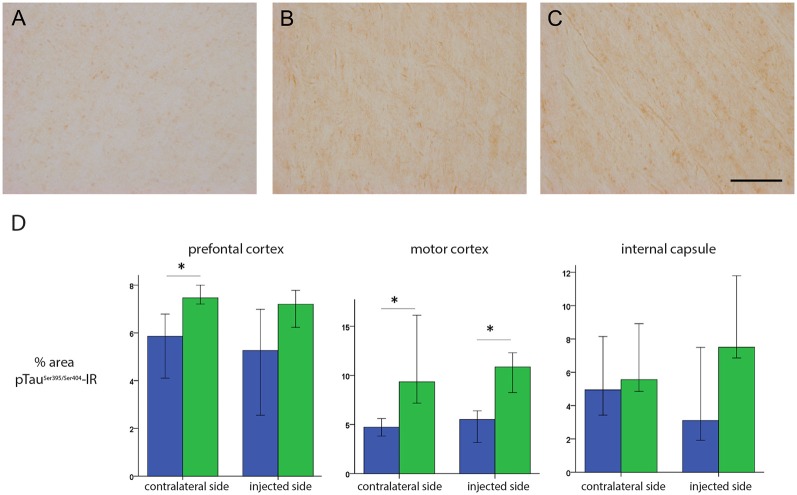
pTau^Ser395/Ser404^ IR following HD-GFP or HD-LRRK2^G2019S^ injection in the SN. Representative images of pTau^Ser395/Ser404^ IR from the frontal cortex of **(A)** an intact animal; **(B)** the contralateral hemisphere and **(C)** the injected hemisphere. **(D)** Quantification of signal intensity from pTau^Ser395/Ser404^ IR in the prefrontal cortex, motor cortex and internal capsule (*n* = 3 monkeys). Scale bar = 200 μm; *p ≤ 0.05.

These data prompted us to compare HD-GFP (a HD CAV-2 vector expressing GFP) and HD-LRRK2^G2019S^ injections in the SN. The results from six monkeys (three received HD-LRRK2^G2019S^ and three received HD-GFP) injected in the left SN are shown. During the 6-month follow-up, we found no notable differences in DTBZ or FDG uptake in HD-LRRK2^G2019S^-injected vs. the HD-GFP-injected animals, or between the injected and non-injected hemisphere (not shown). The monkeys were killed, and the brains were prepared for histology and stereology. While the number of VMAT2^+^ cells was similar in all 12 SNs (i.e., three HD-GFP- and three HD-LRRK2^G2019S^-injected hemispheres, and the six non-injected hemispheres) there was a modest reduction in the number of TH^+^ cells in the injected hemispheres (Table 4). We then compared pTau^Ser395/Ser404^ IR in HD-LRRK2^G2019S^- vs. HD-GFP-injected animals. While, we found no differences in pTau^Ser395/Ser404^ IR in the internal capsule, there was a modest increase in the frontal and motor cortex of HD-LRRK2^G2019S^-injected monkeys (Figure 6). Finally, we found vector genomes in both hemispheres and, as expected, higher levels in the injected hemispheres (Table 5).

**Table 4:** TH^+^ and VMAT2^+^ cells in the SN.

Cohort	Side	TH^+^ neurons mean (SD)	VMAT2^+^ neurons mean (SD)	*p* TH/VMAT2*
HD-LRRK2^G2019S^-SN	Right	1,24,000 (± 16,600)	86,300 (± 6,340)	0.08/0.03
	Left	1,04,000 (± 3,870)	87,300 (± 17,100)	0.03/0.03
HD-GFP -SN	Right	115,000 (± 10,200)	91,700 (± 17,400)	0.03/0.03
	Left	99,500 (± 2,500)	91,600 (± 27,800)	0.03/0.03

*U-Mann Whitney test.

**Table 5:** Vector genomes in the SN-injected cohort.

	Left hemisphere			Right hemisphere		
	SN	putamen	cortex	SN	putamen	cortex
HD-GFP	80,000 (± 22,500)	2,550 (± 1,320)	186 (± 42)	7,450 (± 2,520)	49 (± 260)	65 (± 29)
HD-LRRK2^G2019S^	2,20,000 (± 34,500)	1,270 (± 145)	434 (± 142)	2,840 (± 595)	552 (± 124)	76 (± 40)

Together, these data suggest that following unilateral HD-LRRK2^G2019S^ injection into the SN, LRRK2^G2019S^ increased pTau^Ser395/Ser404^ levels, but did not induce changes in nigrostriatal terminal density or glucose metabolism in the striatum, or the number of VMAT2^+^ cells in the SN.

## Discussion

Similar to the results found in other species (del Rio et al., 2019), we demonstrated here that CAV-2 preferentially infects neurons and is transported to efferent sites when injected into the *M. fascicularis* brain parenchyma. These data are consistent with the neuronal expression of the coxsackievirus and adenovirus receptor (CAR) and its role in axonal transport in rodent neurons (Salinas et al., 2009; Loustalot et al., 2016; Zussy et al., 2016). While little is known concerning CAR in the *M. fascicularis* brain, CAR expression pattern (i.e., in which subtype/population of neurons and in which structure), as well as the monkey neuroanatomical connections, and brain size, likely influenced CAV-2 vector tropism and biodistribution efficacy.

The mean age of PD onset for LRRK2^G2019S^ mutation carriers is 57.5 years (Healy et al., 2008), which is similar to the age of onset of idiopathic PD, and suggest that age-related factors can also play a role in the genesis of symptoms in these patients. In our pilot assays, we used 4–5 years old monkeys, which may have precluded the rapid inception of functional and/or histological LRRK2^G2019S^-associated disease phenotype. Of note, we also used the relatively weak RSV promoter to drive LRRK^G2019S^ expression to better mimic physiological levels, and therefore disease progression. While injections in the putamen led to a lower efficacy of SN infection, they appeared to induce greater impact concerning perturbed striatal metabolic activity and histological factors. Whether these data reflect a greater pathological role for LRRK2^G2019S^ activity in the neurons in the putamen vs. those in the SN, or that the neurodegenerative process could begin in the nigrostriatal projections (Burke and O’Malley, 2013) needs more analyses. However, if this is the case, bilateral putamen injections in aged monkeys will likely allow more robust disease inception. Consistent with this reasoning, the modest reduction of DTBZ uptake in the contralateral putamen may be indicative of the impact of bilateral SN projections and compensatory effects. Interestingly, following HD-LRRK2^G2019S^ putamen injections the changes in DTBZ uptake resembles that seen patients (De La Fuente-Fernández et al., 2003; Ishibashi et al., 2014), and is in contrasts to that seen following MPTP intoxication (Snow et al., 2000; Blesa et al., 2010). While we did not compare DTBZ uptake to putamen injection of HD-GFP, our observations mirror the data from pre-symptomatic carriers of an LRRK2 mutation (Adams et al., 2005; Nandhagopal et al., 2008). The modest reduction in radiotracer uptake by the striatum could be indicative of the preclinical phase of Parkinson’s disease, which is estimated to be an annual reduction of 4.7% (Hilker et al., 2005). We do not know if the decrease in DTBZ uptake in these monkeys would have continued and led to the motor manifestations of Parkinson’s disease.

Importantly, the morphological anomalies we found are similar to reports using other LRRK2 forms (Li et al., 2009; Ramonet et al., 2011), and were not found in the HD-GFP-injected animals. Interestingly, LRRK2^G2019S^ was able to increase the level of pTau^Ser395/Ser404^ in fibers of brain regions that project into the putamen or SN. Tau binds tubulin to stabilize microtubules and promotes tubulin assembly into microtubules. The maintenance of cellular morphology and transport of molecules and organelles over long distances depends on microtubules stabilization by tau in neurons and altered tau function could block the transport of organelles, neurofilaments, and vesicles (Spires-Jones et al., 2009). Our results agree with those reported by Melrose et al. (2010) who described a similar increase in pTau^Ser395/Ser404^ IR in white matter fibers in the thalamus, hypothalamus, striatum, and midbrain, as well as tracts in the pontine base and medulla of LRRK2^G2019S^ mice. Finally, 21–54% of LRRK2-associated Parkinson’s disease patients do not show apparent Lewy bodies in the SN although they show loss of dopaminergic neurons in this area (Poulopoulos et al., 2012; Kalia et al., 2015). Also, higher grade tau pathology in cortical areas is a prominent feature of LRRK2-associated Parkinson’s disease (Henderson et al., 2019).

In summary, our study demonstrates that CAV-2 vectors are powerful tools for gene transfer to the *M. fascicularis* brain and that, following optimization, CAV-2–mediated expression of LRRK2^G2019S^ may be used to induce a functional impairment of the nigrostriatal dopaminergic system inducing histological changes.

## Data Availability Statement

The datasets generated for this study are available on request to the corresponding author.

## Ethics Statement

The animal study was reviewed and approved by Ethics Committee for Animal Experimentation of the University of Navarra.

## Author Contributions

RL and EK: study concept, design, and supervision. AG and EK: design and production of viral vectors. CC and I-CM: primates surgery. CC and IM: behavior test and data analyses. CC, IM, and MC-A: PET scan images acquisition and analysis. CC, I-CM, IM and MH: acquisition, analysis, and interpretation of histological data. IM-V: DNA extraction and PCR data analysis. CC: statistical analyses. IM-V and CC: initial draft of the manuscript. EK and RL: critical revision of the manuscript.

## Conflict of Interest

The authors declare that the research was conducted in the absence of any commercial or financial relationships that could be construed as a potential conflict of interest.
